# Prevalence and factors associated with child marriage, a systematic review

**DOI:** 10.1186/s12905-023-02634-3

**Published:** 2023-10-10

**Authors:** Asma Pourtaheri, Seyedeh Belin Tavakoly Sany, Monavvar Afzal Aghaee, Hamideh Ahangari, Nooshin Peyman

**Affiliations:** 1 Department of Health Promotion and Education, School of Health, Mashhad University of Medical Sciences, Mashhad, Iran; 2 Department of Health, Safety and Environment management (HSE), School of Health, Mashhad University of Medical Sciences, Mashhad, Iran; 3 Department of Epidemiology and Biostatistics, School of Health, Mashhad University of Medical Sciences, Mashhad, Iran

**Keywords:** Marriage age, Prevalence, Child, Systematic review

## Abstract

**Background:**

Girl child marriage is increasingly recognized as a critical barrier to global public health and gender discrimination. There are still more gaps in the global rate of child marriage and the underlying factors. Thus, the present systematic review aimed to explore the prevalence of child marriage and the underlying factors.

**Methods:**

A comprehensive search was conducted for all English-language studies that measured the prevalence of child marriage and its correlates from 2000 to March 2022, indexed in PubMed, Scopus, Web of Science, Poplin, and Google Scholar databases. Child marriage is defined as marriage under the age of 18. In the present study, Joanna Briggs' quality assessment checklist was used for data collection. Two independent reviewers reviewed all the articles.

**Results:**

In total, 34 eligible prevalence articles and 14 trend articles were included in the study with data from 127,945 participants. The prevalence of child marriage ranged between 1.8% to 90.85%. In most studies, the trend of child marriage was decreasing. The most important individual factors include the respondent's education and occupation, interpersonal factors such as the education and occupation of parents and husband, family size and type. Community factors include socioeconomic status, region, residence, ethnicity, and religion at the social level.

**Conclusion:**

Despite a central focus of research and policies on interventions that decrease child marriage, this phenomenon is still prevalent in many places. Therefore, further specific interventions are required to improve education, reduce poverty and inequality. This may help achieve the 2030 Sustainable Development Goals.

**Supplementary Information:**

The online version contains supplementary material available at 10.1186/s12905-023-02634-3.

## Background

The United Nations Children's Fund (UNICEF) defined Child Marriage (CM) as marriage occurring at the age younger than 18 years [1]. Nearly 15 million girls under the age of 18 marry around the world annually [2]. A total number of 750 million women get married during childhood nowadays, and this number will be as large by 2030 unless a significant change is introduced, [3].

CM is a major health and child rights issue in many low- and middle-income countries [4–6]. Human rights experts argue the practice violates children's human rights and threatens their health and development [7]. Much human rights evidence, over the past few years, has dealt with child marriage, such as the UN Convention on Consent to Marriage, Minimum Age of Marriage and Registration of Marriages (1962) [8], Convention on the Elimination of All Forms of Discrimination against Women (1979) [9], Convention on the Rights of the Child (1989) [10]. According to the Convention on the Rights of the Child, one criterion for international development is the percentage of women population married before the age of 18 (10). In addition, the percentage of women married before the age of 18 is included in the annual report on the achievement of the UN Sustainable Development Goals [11].

The growing focus on CM as a global development issue also appears to reflect growing concern over its potential impact on the population health. United Nations Children’s Fund (UNICEF), the World Bank, the United Nations Population Fund (UNFPA) consider the potential adverse effects on health a great concern [12, 13].

Girls who marry under the age of 18 come to have children at an early age [3, 14]. Studies have shown that they have less control over their fertility. For example, they have less access to contraception, have more unwanted pregnancies, have higher chances of terminating pregnancies, and have shorter intervals between childbirths [15, 16]. As a result, they are at a higher risk of obstetric complications, known as the leading cause of mortality among adolescents in low- and middle-income countries [17, 18]. Girls married early are more likely to experience domestic violence [19, 20]. The risk of sexually transmitted diseases (STD) increases in this population [21, 22]. Also, early marriage affects girls’ psychological well-being [23, 24].

Several studies have explored the correlates of child marriage. Child marriage is common among poor families because there is little motivation or resource to invest in future [25]. The loss of educational opportunities, unemployment are related to poverty [26] and natural vulnerabilities also reduce economic functioning, thus negatively affecting poor households [27]. Social, religious, cultural and traditional beliefs and norms play a major role in the continuation of early marriage [28].

An essential step to achieve the fifth goal of the Sustainable Development Goals 2030 (SDGs), concerning the elimination of all forms of violence against women, entails the preventative interventions for child marriage. However, this act of elimination by 2030 will require a substantial acceleration, equivalent to a 23% reduction [29].

Despite the growing literature on strategies and methods to reduce child marriage [30–32], there is no systematic review on the prevalence of child marriage and the underlying factors. In a systematic review, Zaman reported that about 1% of 15- 19-year-old population in Canada were in common-law unions or married in 2016 and there was no national estimation of the frequency of child marriage [33]. In a review of rural residence, Nasser Subramanee introduced low education level, poor economic background, low exposure to mass media and religion as the underlying factors of child marriage [34].

A combination of child marriage with political, economic and religious matters has led to inaccurate statistics in many countries, which challenges preventive policies. Comprehensive information helps understand the prevalence rate of child marriage in different contexts and provides a perspective for policymakers to develop appropriate intervention strategies. In addition, recognition of the main influential factors underlying child marriage could contribute to systematic interventions against these factors. Therefore, the objectives of the present study were: 1) to systematically review the prevalence of child marriage in different parts of the world 2) to represent trends in child marriage over time 3) to recognize the factors that affect child marriage.

## Methods

### Design of study

In the present review, the Preferred Reporting Project for Systematic Review (PRISMA) was used along with the PICO framework to explore the prevalence of child marriage and the underlying factors [35]. Review techniques were used to address the following research questions.

1. What is the global prevalence of child marriage?
2. What are the trends in child marriage in different parts of the world?
3. What factors account for child marriage?

### Information sources and search strategy

The prevalence of child marriage among married women was explored from January 2000 to March 2022 in five databases, including Scopus, PubMed, Web of science, Poplin and Google Scholar to review the related gray literature.

According to Haddaway [36], most gray literature appears in the first 200 citations provided by Google Scholar. Haddaway suggests that the reviewers focus on the first 200–300 records. As suggested, the first 300 records were retained and sorted by relevance.

The search terms included Medical Subject Headings (Mesh), free words, and selected keywords. The keywords included "early marriage", "teenage marriage", “prevalence”, “Incidences”, "Incidence Proportion", "Cumulative Incidence", "Incidence Rate". The main components were combined by Boolean operators (AND, OR) in the search strategy.

Here is an instance of search strategy in PubMed:

- (((((("early marriage"[Title/Abstract]) OR ("spouse child"[Title/Abstract])) OR ("teenage marriage"[Title/Abstract])) OR ("adolescent marriage"[Title/Abstract])) OR ("child bride"[Title/Abstract])) OR ("forced marriage"[Title/Abstract])).

AND

- ((((((((Prevalence[Title/Abstract]) OR (Incidences[Title/Abstract])) OR ("Incidence Proportion"[Title/Abstract])) OR ((Proportion[Title/Abstract] AND Incidence[Title/Abstract]))) OR ("Cumulative Incidence"[Title/Abstract])) OR ((Incidence[Title/Abstract] AND Cumulative[Title/Abstract]))) OR ("Incidence Rate"[Title/Abstract])) OR ((Rate[Title/Abstract] AND Incidence[Title/Abstract]))).

Finally, we came across additional studies through a manual search of references.

### Selection process: inclusion and exclusion criteria

The PICO-SD guidelines were followed, including patients, interventions, comparisons, outcomes, and study design to extend the criteria. The inclusion criteria were married women under the age of 18, and a report of the prevalence of child marriage. As part of our supplementary information, the details of inclusion and exclusion criteria are summarized in Table S1.

Letters to the editor, intervention studies, qualitative studies, reviews, case reports, meeting summary reports, and studies that did not report the sample size were excluded. According to the UNICEF, child marriage is defined as marriage under the age of 18.

### Identification and selection of studies

All potentially relevant studies in each database were imported into electronic reference management software (EndNote X8.8) and spreadsheets (Excel 2016). Article selection for the present review was done in several steps by two independent researchers. In the first step, duplicate studies were eliminated. In the second step, the title and abstract of each study were screened according to some predefined inclusion and exclusion criteria. The articles that did not meet the inclusion criteria were removed. To determine eligibility, the full text, methods, and results sections were read. In the present review, only the articles that were totally agreed to be included by both reviewers were included. When there was a disagreement about the eligibility of an article, it was resolved by referring to the third researcher.

### Data extraction

Two independent authors (A.P and B.T) extracted data from studies through parallel processes.

Through discussion or consultation with the third and fourth reviewers, any doubts and disagreements between the authors (M.A and N.P) regarding the data extraction were resolved.

For the independent double data extraction, a predesigned table was used to suit systematic reviews and extract the following information. The data were extracted according to the purpose of study: (1) authors/publish year, country of origin, type of participants, total papulation, sample size, girls married (number of adolescents who got married under the age of 18), marriage age range, prevalence of child marriage (2), and factors underlying child marriage. The results of all studies were collected according to the purpose of study and research question. A total number of 28 eligible articles and 14-trend articles were included in the study. In addition, 6 studies were added by searching in the reference list of articles. Finally, 34 scientific papers and 14 trend articles were included.

### Risk of bias assessment

Joanna Briggs' Quality Assessment Checklist was used for Cross-sectional Analytical Studies [37]. This checklist contains eight questions. Two questions were not used in the present study, as they were objective in type. They explored whether standard criteria were used to measure the condition, and whether the outcome behavior was measured in a valid and reliable way. Thus, the quality of the studies was evaluated with 6 questions.

### Extraction of the prevalence of child marriage

In 28 of the overall 34 articles, the prevalence of child marriage was stated directly [38–65], and it was considered as the prevalence rate. However, in five articles, the prevalence was not mentioned directly [66–70]. Thus, the prevalence was estimated as the proportion of the girls married under 18 years to the total population.

### Evaluation of trend studies

The Average Annual Rate of Reduction (AARR) was computed using the following equation to check relative trends:

1 $$\mathrm{AARR}(\%)=\frac1{years\;between\;survey}\times\mathrm{In}\;\left(\frac{prevalence\;at\;second\;survey}{prevalence\;at\;first\;survey}\right)\;\times100$$

AARR in Eq. (1) represents the average relative decline in child marriage each year.

SDG target 5.3 refers to the "Elimination of early childhood and forced marriage" by 2030.

To assess whether countries are on track to achieve this goal, the prevalence of EM in 2030 was anticipated for each country using the following equation:

2 $$2030\;\mathrm{prevalence}\left(\%\right)=\mathrm{prevalence}\;\mathrm{at}\;\mathrm{second}\;\mathrm{surevey}\times{(1-(0.01\times\mathrm{AARR}))}^{\mathrm n}$$

The AARR for each country is calculated using formula (1), and *n* represents the number of years between a country's most recent survey and 2030 [71].

### Analysis

Extensive data on the prevalence of child marriage needed to summarize the studies descriptively and evaluate them qualitatively. Therefore, no meta-analysis was done. It was very intuitive and practical to combine the results into categories of related factors, such as education, socioeconomic status, and parental conditions. These categories emerged from the data and were not predetermined.

## Results

### Search outcome: study design

**Fig. 1:**
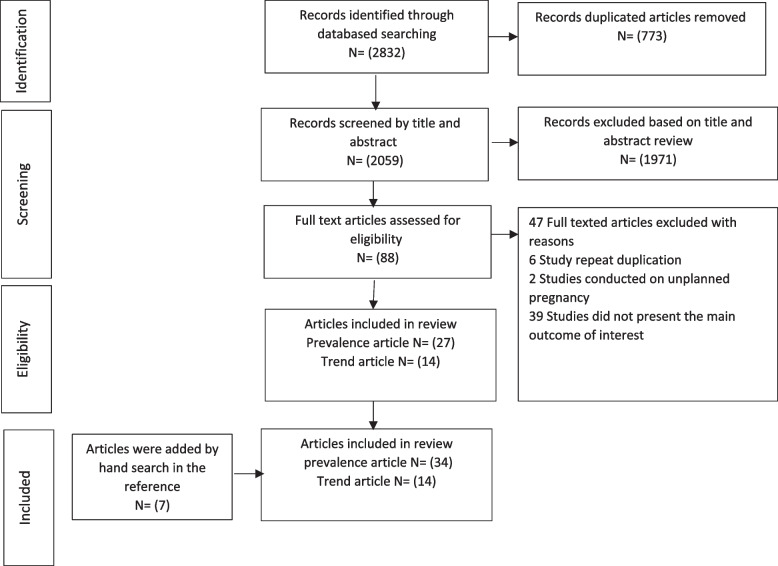
PRISMA flow chart diagram describing selection of studies for systematic review on Prevalence and factors relating of child marriage

The systematic review included 32 prevalence articles [38–50, 52–70, 72], two theses [40, 51] with data from 127,945 participants, Of these participants, 82,147 were married under the age of 18 and there were also14 trends articles [71, 73–85] (Fig. 1, Table 1).

**Table 1:** Summary characteristics of cross sectional studies included in the systematic review

	**Author/year**	**Country**	^a^**Income**	**Type of participant**	^b^**Total population**	^c^**Girls married**	**Marriage Age range**	**Prevalence of child marriage (%)**
1	Rahman 2005 [38]	Bangladesh	Lower-middle	Adolescents aged 10–19 years	3362	2256	16.2 ± 1.9	67.1
2	Raj/ 2009 [72]	India	Lower-middle	Married—aged 20–24 years	22 807	7730	< 18	44.5
3	Nasrin/ 2012 [39]	Bangladesh	Lower-middle	Married women	609	510	< 18	83.7
4	Adams/ 2013 [40]	Nigeria	Lower-middle	Adolescents 10–19 years and parents	397	17	16.1 ± 2.3	11
5	A Ali 2014 [41]	Sudan	Low	Ever married women aged ≤ 35 year	1700	781	15.4	45.9
6	Al Ridhwany 2014 [42]	Iraq	Upper-Middle	Married women in child-bearing age having at least two living children	1302	204	18.5 ± 4.2	15.7
7	Asrese 2014 [43]	Ethiopia	Low-Income	Currently married or ever married	476	395	< 18	83
8	Dharan. 2014 [44]	Nepal	Lower-Middle	Women aged 15 to 45 years	300	208	< 18	69.3
9	Sumon 2014 [45]	Bangladesh	Lower-Middle	Married aged less than 50 year	600	538	15–19	89.7
10	Ghrayeb/ 2015 [46]	Palestinian	-	Rural married women	500	207	< 18	41.4
11	Envuladu 2016 [47]	Nigeria	Lower-Middle	Secondary School Girls Within The Ages Of 10-25 years	4013	74	< 18	1.8
12	Islam 2016 [48]	Bangladesh	Lower-Middle	Ever-married women aged 12–49 years	17,808	13,837	< 18	**77.7**
13	Hamed/ 2017 [49]	Egypt	Lower-Middle	Ever-married women aged 20–60 years	1064	640	17.86 ± 3.27	60.15
14	Mpilambo, 2017 [66]	Congo	Low	Young women aged 15–24 years	3481	2238	16.7	64.29
15	Rahman 2017 [50]	Bangladesh	Lower-Middle	Married women aged 15 to 49 years	16,830	12,740	< 18	75.04
16	Groot 2018 [67]	Ghana	Lower-Middle	Ever-married women aged 20–29 years	1943	509	< 18	26.19
17	Pham 2018 [51]	Viet Nam	Lower-Middle	Girls from young	424	55	< 18	12.8
18	Basazinewu/2018 [55]	Ethiopia	Low	women's of 15–50 years	350	318	< 17	90.85
19	Rumble 2018 [70]	Indonesia	Lower-Middle	women aged 20 to 24	6578	938	15.82 ± 0.05	14.27
20	Ahonsi 2019 [52]	Ghana	Lower-Middle	women aged 20–24(GDHS)	1613	334	17.7	20.68
21	Bezie/ 2019 [53]	Ethiopia	Low	married women aged 15–49 years	373	167	17 ± 3.2	44.80
22	Gashaw 2019 [54]	Ethiopia	Low	married women age 15–49 year	9262	5942	< 18	64.2
23	Kalum 2019 [56]	Congo	Low	women of childbearing age	5304	1227	12–18	23.1
24	Alem/ 2020 [68]	Ethiopia	Low	all women aged from 15– 49 years	11,646	7322	< 18	62.87
25	Chowdhury 2020 [69]	Bangladesh	Lower-Middle	-	8,699	7,046	< 18	80.99
26	Manandhar/ 2020 [60]	Nepal	Lower-Middle	Married women age less than 50 years	358	187	17.2	52.2
27	Talukder 2020 [61]	Bangladesh	Lower-Middle	married women aged 15–49	17,883	10,551		59
28	Tekile/2020 [62]	Ethiopia	Low	all female community members	1120	544	15	48.57
29	Berliana 2021 [57]	Indonesia	Lower-Middle	reproductive women aged 15– 49 years	7207	1009	15–19	14
30	Hasanah 2021 [58]	Indonesia	Lower-Middle	married women	1687	909	< 18	53.9
31	Roy 2021 [59]	Bengal	Lower-Middle	married women 15–49 years	357	230	< 18	64.42
32	Aychiluhm/2021 [63]	Ethiopia	Low	All women aging 15–49 years	2887	2098	< 18	73
33	Bengesai/2021 [64]	Zimbabwe	Lower-Middle	Ever-married women aged between 20–29 years	2380	821	24.2 ± 2.7	36.8
34	Masresha 2021 [65]	Ethiopia	Low	adolescent women between the ages of 15 to 19	597	232	17.62 ± 1.36	38.9

^a^This information was followed linked; https://datatopics.worldbank.org/world-development-indicators/the-world-by-income-and-region.html

^b^Total papulation that was examined in these studies

^c^Total girls that got married under 18 year

A study reported the phenomenon of child marriage among Syrian refugees. This study was excluded due to the high rate of child marriage in this population and the potential bias in results [86].

The sample size ranged between 300 [44] and 22 807 [72]. The studies included were published between 2005 and 2021, but they were mostly published in 2019–2021 (*n* = 15, 44.11%). In total, the data were collected from 14 countries. In terms of geographic distribution, 15(44.11%) of the included articles had been conducted in South and Southeast Asia [38, 39, 44, 45, 48, 50, 51, 57–61, 69, 70, 72], 16 in Africa (47.05%) [40, 41, 43, 47, 52–56, 62–68], and three in the Eastern Mediterranean (8.82%) [42, 46, 49]. Two countries, Bangladesh (*n* = 7) [38, 39, 45, 48, 50, 61, 69] and Ethiopia (*n* = 8) [43, 53–55, 62, 63, 65, 68] accounted for approximately half of the studies included in the present review.

Overall, the majority of studies (*n* = 33, %97.05) were conducted in low- and middle-income countries [87]. All included studies followed a cross-sectional design. About 47% (*n* = 16) of studies relied on data from the National Survey and the Demographic Health Survey (DHS) [48, 50–52, 54, 57, 58, 61–64, 66–69, 72].

### Risk of bias assessment

Four studies met the inclusion criteria. Only one study used a valid and reliable method to measure exposure. Sixteen studies identified confounders. Eleven described details of the setting and subjects. Strategies to control the confounders were adopted in 26 studies.

Appropriate statistical analyses and valid ways to measure outcomes were used in 26 studies. More details of the quality assessment of articles are shown in Table S2.

### Prevalence of child marriage

Most articles (*n* = 31,91.17) aimed to show the prevalence and determinants of child marriage, and two (5.88%) focused on knowledge about and attitudes towards child marriage [38, 40]. In the present systematic review, there was considerable variation in the prevalence of child marriage. Different estimates of the prevalence of child marriage were reported in the same country at the same time. Instances are Bezie [53] and Gashaw [54] from Ethiopia (2019), Berliana [57] and Hasanah [58] from Indonesia (2021). The reported prevalence of child marriage ranged from 12.8% [51] to 89.7% [45] in Asia 1.84% [47] to 90.85% [55] in Africa and 15.7% [42] to 60.15% [88] in the Mediterranean.

### Trends in child marriage

Fourteen trend papers were found among national studies [71, 73–82, 84, 85]. More than 78.50% (*n* = 11) of the articles reported child marriage trends in Asia [71, 73, 74, 76–82, 85]. Two articles were conducted in Africa (14.28%) [83, 84] and one in the United States (7.14%) [75].

The trend of child marriage has been declining in most studies (*n* = 12, 85.71%), with an exception of Iran, where the prevalence of child marriage increased from 10.27 to 11.21 in 2006–2016 (AARR = 0.87) [80] and China, where the prevalence of child marriage increased from 2.41 to 2.85 in 2000–2010 (AARR = 1.67) [85]. The highest decrease was observed in India in 2016–2006 (AARR = -3.63) [71] and the lowest decrease was in West Africa in 2014–2006 (AARR = -0.58) [83].

The prevalence of child marriage in 2030 was estimated using a formula based on the prevalence reported in the study. Bangladesh is predicted to have the highest number of child marriage followed by the United States [75] (Table 2).

**Table 2:** Studies of child marriage trends included in systematic reviews

Author	Continent	Country	Year	Duration Time	Prevalence Of CM (%)	^a^AARR	Prevalence Of CM 2030 (%)
Moor/2009 [73]	Asia	India	1992–2006	14	50–45	-0.74	35.1
Marshan/2013 [74]	Asia	Indonesia	2001–2010	9	18.2–13.5	-3.31	10.26
Marashi/2017 [75]	American	United States	1992–2013	21	1.1–0.8	-1.49	0,64
Modak/2018 [76]	Asia	India	2011–2016	5	31.88 -26.8	-3.47	22.78
Biswas/2019 [77]	Asia	Bangladesh	2004–2014	10	69–52	-2.82	42.12
MacQuarrie/2019 [78]	Asia	Bangladesh	1993–2011	18	41–20	-3.58	11.4
		India	1998–2016	18	64–45	-1.76	37.35
		Indonesia	1991–2012	21	50–37	-1.41	29.6
		Nepal	1996–2016	20	75–55	-1.55	44
Wahyudi/2019 [79]	Asia	Indonesia	2000–2014	14	2.65–1.96	-2.14	1.41
Kumar/2020 [81]	Asia	India	1993–2011	18	87–77	-0.61	63.91
Paul/2020 [82]	Asia	India	1992–2016	24	54.2–26.8	-2.88	16.34
Azimi/2020 [80]	Asia	Iran	2006–2016	10	10.27–11.21	0.87	9.64
Scott/2021 [71]	Asia	Bangladesh	2007–2014	7	77–69	-1.55	64.17
		Nepal	2005–2016	11	63–52	-1.72	46.28
		India	2006–2016	10	59–41	-3.63	29.93
		Pakistan	2008–2018	10	50–37	-3.01	25.67
Fatusi/2021 [83]	African	West Africa	2006–2014	8	43.5 -41.5	-0.58	39.84
Sagalova/2021 [84]	African	West And Central Africa	1990–2010	20	37.3–24.9	-2.02	16.43
Fan/2022 [85]	Asia	China	2000–2010	10	2.41–2.85	1.67	2.30

^a^Average Annual Rate of Reduction

### Related factors to child marriage

The factors that influence individuals at a personal level can contribute to a girl being more likely to marry at a young age. Interpersonal factors that increase the likelihood of a girl getting married at a young age are influenced by her relationships with family members, peers, and teachers. The level of presumption has an impact on a girl's immediate social circle, which consists of her family members, peers, and schoolmates. These individuals play a role in shaping her behavior and overall experiences. The influences at the community level refer to the factors that raise the risk level based on the community and social surroundings, particularly schools and neighborhoods. Societal level influences refer to broader factors that have an impact on child marriage. These factors include religious or cultural beliefs, and societal norms that contribute to or maintain disparities between different groups of people.

We categorized the factors related to child marriage based on the Social Ecological Level, which consists of four levels that influence a child's life: the individual, interpersonal, community, and societal levels. The factors that influence individuals at a personal level can contribute to a girl being more likely to marry at a young age. The interpersonal level influences are factors that increase the risk of a girl getting married early because of her relationships with family members, peers, and teachers. The level of presumption influences a girl's closest social circle, which includes family members, peers, and school partners who shape her behaviors and experiences. The influences at the community level refer to the factors that raise the risk level based on the community and social surroundings, particularly schools and neighborhoods. Societal level influences refer to broader factors that have an impact on child marriage. These factors include religious or cultural beliefs, societal norms that contribute to or maintain disparities between different groups of people [89].

Individual factors related to child marriage include the low education of respondents [38–45, 48–50, 52–54, 56–63, 65, 66, 68–70, 72], unemployment of respondents [38, 45, 48, 54, 66], limited knowledge about the complexities of marriage, pregnancy, and marriage laws [38, 43, 49, 62, 65], and engaging in sexual activity before the age of 17 [64, 66]. Interpersonal factors related to child marriage include the husband's low education [39, 41, 42, 44, 45, 48, 50, 53, 54, 61, 64, 68–70, 72], the husband's occupation as a worker or farmer [44, 50], low parental education [41, 42, 47, 53, 59, 65], parents working as farmers [47, 56] or low-skilled jobs [41, 42], having a family size of more than 5 [41, 47, 49, 50, 70], and belonging to a nuclear [45], or extended [42] family type., decision-making by parents [38, 44, 63, 68], consanguineous marriage (marrying close relative) [42, 49], Community factors related to child marriage include low socio-economic status [39, 44, 48–54, 56–59, 61–63, 66, 70, 72], rural residence [41–43, 48–52, 54, 57, 62–64, 66, 69, 70, 72]. region (regional division in a country) [47, 48, 50–52, 54, 61, 66, 68, 72], Kurdish, Arab [42], Dalit [44], Gurma [52], Sundanese [58], Foreign/Non-Congolese and Cuvette central ethnicity [66], Additionally, the lack of access to media is also a significant factor [43, 45, 54, 57, 65, 66, 70] and Societal factors related to child marriage include Islam [39, 47, 50, 52, 54, 69], Hinduism [60, 72], Orthodox [54] religions, and no religions [52]. Factors associated with child marriage are shown in Tables 3 and 4.

**Table 3:** A summary of factor associated with child marriage in the article

**Articles**	**Education of Respondents**	**Socio-economic status**	**Residence**	**Husband's education**	**Religion**	**Region**	**Ethnicity**	**Access to Media**	**Parental education**	**Respondent's Job**
1	*									*
2	*	*	*	*	*	*				
3	*	*		*	*					
4	*									
5	*		*	*					*	
6	*		*	*			*		*	
7	*		*					*		
8	*	*		*			*			
9	*			*				*		*
10										
11					*	*			*	
12	*	*	*	*		*				*
13	*	*	*							
14	*	*	*				*	*		*
15	*	*	*	*	*	*				
16										
17		*	*			*				
18										
19	*	*	*	*				*		
20	*	*	*		*	*	*			
21	*	*							*	
22	*	*	*	*	*			*		*
23	*	*								
24	*			*		*				
25	*		*	*	*					
26	*				*					
27	*	*		*	*	*				
28	*	*	*							
29	*	*	*					*		
30	*	*					*			
31	*	*							*	
32	*	*	*							
33			*	*	*					
34	*							*	*	

**Articles**	**Knowledge**	**Family size**	**Parent's occupation**	**Family Type**	**Parental decision making**	**Consanguineous marriage**	**Age of first sex**	**Husband job**	**Others**
1	*				*				
2									
3									
4									
5		*	*						
6			*	*		*			*
7	*								
8					*			*	
9				*					
10									
11		*	*						*
12									
13	*	*				*			
14							*		*
15		*						*	
16									
17		*							
18									
19		*							
20									
21		*							
22									
23			*						
24					*				
25									*
26									
27									
28	*								
29									
30									
31									
32					*				
33							*		*
34	*

**Table 4:** Determinants of child marriage in the social ecological framework

Summary of Findings	Determinants	Social Ecological Level
Girls with lower levels of education are more vulnerable to early marriage	Education of Respondents	**Individual level**
Girls who do not work are often married at a young age	Respondent's Job	
Participants' awareness and literacy levels regarding the consequences of early marriage, pregnancy, and the legal age are related to early marriage. Awareness of these factors can be associated with a reduction in early marriage	Knowledge	
Girls who have their first sexual experience before the age of 17 are more likely to marry at a young age	Age of first sex	
Men with low levels of education are more likely to marry underage girls	Husband's education	**Relationship level**
Men who worked as farmers and laborers were more likely to marry young girls compared to men in other occupations	Husband job	
Parents with low education often see their daughters marrying at a young age	Parental education	
Child marriage was more common among parents who were farmers or had jobs that required little skill	Parent's occupation	
Child marriage is more common in large families with more than four children	Family size	
Child marriage is more common in nuclear and extended families	Family type	
Young girls often lack the authority to make decisions regarding their own marriage, as it is typically determined by their families	Parental decision making	
Younger girls are more likely to marry their relatives	Consanguineous marriage	
Low socio-economic status encompasses factors such as family income, social class, wealth index, and overall socio-economic status. If a family has a low socio-economic status, the likelihood of early marriage increases	Socio-economic status	**Community level**
Girls marry more frequently in rural areas	Residence	
The division of regions in a country can affect child marriage, for example, the north and south. Child marriage is more prevalent in underdeveloped regions	Region	
Child marriage is more common among Kurdish, Arab, Gurma, Agew, Sundanese, Foreign/Non-Congolese, and Cuvette Central ethnicities	Ethnicity	
Girls who lack access to media are more likely to marry at a young age compared to girls who have access to media	Access to Media	
Child marriage is most prevalent among Muslims. This practice is also observed among Hindus, Orthodox, Protestant and non-religion	Religion	**Societal level**

## Discussion

Child marriage adversely affects children and society in many ways. The development of interventions depends on access to comprehensive information on the prevalence of child marriage and its geographic distribution. Thus, the purpose of the present study was to measure the prevalence of child marriage, the associated factors, and the trend of child marriage.

Ethiopia was found to have the highest rate of child marriage in 2018 (%90.85) [55]. Ethiopia is among the countries marked by the highest rate of early marriage in the world, with one in two girls married before the age of 18 and one in five married before the age of 15. It can be due to the acts of discriminating girls by parents, adhering to the traditions and norms of society, leaving natural gifts behind after marriage, forming a social bond (kinship), maintaining virginity and preventing sex before marriage, purchasing dowry (for financial benefits) [55].

In 2016, Nigeria had the lowest prevalence of child marriage (%1.8) [47]. This study measured the prevalence of child marriage among high school girls. Overall, female students are less likely to marry, and those who get married lose the opportunity to continue education because of the house chores and child care-taking tasks.

The trend of child marriage in India has declined significantly (AARR -0.61 to -3.63). The improvement in women's education and the wealth of married couples during the past decade can be a major reason. The furtherance of women's education in India reduced child marriage by 38% [71].

However, the trend of child marriage seems to be increasing in Niger, Nigeria and Côte d'Ivoire. Living in rural areas, low education and poverty were the foremost factors affecting child marriage in these regions. In addition, in these countries, planning to reduce child marriage seems to be scarce and these countries fail to apply an accurate evaluation mechanism [83].

In two studies in Iran and China, the trend of child marriage was increasing. In Iran, the trend has decreased in the age group of 10–14 years, but has increased in the age group of 15–19. It should be interpreted with caution because distinguishing age groups can overestimate the prevalence of child marriage. However, changes in demographic policies should not be ignored [80]. Child marriage is more prevalent in China's western provinces (Yunnan, Xinjiang, Qinghai, Ningxia, Tibet, and Guizhou). The population of women, autonomous ethnic minorities and poverty is the most important underlying reason [85].

### Related factors

A girl's education was the major individual factor associated with child marriage. Education promotes girls' awareness of all aspects of reproductive health and the negative outcomes of pregnancy [53, 58, 62, 70, 90]. Educated girls are better aware of their rights and can make the right decision about their lives [41, 57, 63, 66, 88, 90]. They spend time studying [47, 54] and have the opportunity to land a job to delay marriage [49, 59]. Education not only prevents girls from getting pregnant and sick, but also improves their social and economic status, allowing them to live in cities [45]. Educated girls can contribute to the health and well-being of their families, and they tend to marry men of their own age [60]. Unavailability of education for any reason including insurgency and armed conflict increases the likelihood of child marriage [66]. In parts of Ethiopia, social norms are so strong that awareness fails to prevent child marriage. Virginity assurance, access to resources and safe future are the most important norms in this country to encourage child marriage [43, 63].

Age of starting sex was associated with early marriage. Increased libido in young people around the world can possibly explain this issue [64, 66].

Education also plays an important role among the interpersonal factors associated with child marriage. Low education also affects parents' understanding of the nature and purpose of marriage, economic factors, environmental factors, personal desires, religious factors, early marriage culture, and adolescents' perception of free sex [58]. Farmer parents usually have low education and socio-economic status, which can be a reason for their daughters’ early marriage [47, 56]. Husband's education is also effective in child marriage. Girls who marry early mostly have uneducated husbands. Generally, men tend to marry women who are less educated (than themselves), and as a result, average-educated men choose women with an average or below-average level of education, which eventually leads to early marriage [50]. Increasing men's awareness of the adverse effects of early marriage can reduce the rate of marrying girls under 18 years [61]. Husband's occupation also affects children's marriage due to the education level. Manual workers and farmers marry children because of their low education level [50]. Family size and type can also affect child marriage. Child marriage has been reported in large families [51, 53], and nuclear families [45] both, so it is difficult to establish the relationship between family type and child marriage. Yet, there is evidence to support the finding that households of more than 5 are associated with child marriage [41, 47].

At the community level, various factors were found to be related to child marriage, with one of them being socioeconomic status.. In societies with a low socio-economic status such as India [72], Bangladesh [39, 48, 50, 61], Ghana [52], Ethiopia [53, 54, 62], and Indonesia [57, 70], early marriage reduces financial burden, increases income and improves economic conditions. Adolescents from poor families do not have jobs, and parents with low income cannot afford the living expenses of young girls. In these conditions, girls are considered as a burden, and with marriage, the family's economic pressure is lowered. In fact, in these families, child marriage has been considered an economic strategy [38, 50, 52–54, 59, 70].

Sometimes, girls' stubbornness, curiosity or materialistic desires lead to marriage [52]. In Ethiopia (2021) new brides provide income for their families mainly through "macha" (money and livestock paid by the groom's family). In poor families, the loss of educational opportunities [57, 59], and low parental awareness of the adverse effects of early marriage increase child marriage [50]. Poverty is exacerbated in areas prone to natural disasters, making it difficult for the poor to pay their dowries. In these circumstances, parents' concern for girls' safety increases [48]. Regional as well as religious differences can also account for the rate of early marriage [90].

Research evidence shows that the place/region of residence also affects child marriage as it sets the stage for education, awareness, job opportunities, required amenities, access to mass media, formation of norms and beliefs in people, poverty, natural disasters, differences in urbanization, and religion. Differences in socioeconomic development across regions have led to growth in education [47], and increased access to the internet, media and information [50, 51]. Girls in developed and urban places of residence tend to delay marriage as most of them have higher education and more job opportunities than peers in less developed places [48, 50, 51, 57]. In rural areas, women may not adequately perceive the impact of marriage on their health, education, economy, and even their rights [63]. What's more, the socio-economic status and traditional norms differ across geographical regions, which can encourage child marriage [50].

Ethnicity affects child marriage through cultural norms and education level. In Arab nations, cultural norms promote child marriage [42]. Parents' decision to marry children under the influence of traditions is another factor affecting child marriage. In Nepal, where the Sundanese have the highest number of child marriages, the traditional customs and cultural factors of these ethnic groups make parents responsible for deciding on the right man to marry; thus, girls do not have the right to make independent decisions [58].

At the societal level, religion was the sole factor associated with child marriage. Child marriage is more prevalent in certain religions, such as Islam [39, 47, 50, 69] and Hinduism [60, 72]. There is no clear answer to how religion affects child marriage. The classic interaction of religion and culture [91] and the absence of a legal age for marriage [92] can increase the rate of child marriage. Instances of sociocultural norms [68] are the priority of marrying a family's eldest son in Bangladesh [50], virginity of girls in Ghana [52] and Ethiopia [68], the desire for normative structures such as kinship and extended family [54], family desire to maintain a good name [93, 94], stigmatizing single girls [95] and limited right of decision-making [96]. As a result of this rigid marriage tradition, it forces young women to marry at a young age.

### Limitations of study

There are several limitations in the present study. The first point to consider is that the search was for English-language articles only. Thus, if an article was published in other languages, it was not retrieved in the present review. Second, there is a risk of generalizing the present findings because most articles are from low-income countries. Third, the extensive data made it hard to perform a meta-analysis; thus, it was decided to review the studies qualitatively. Fourth, the present search originally aimed to find the prevalence of child marriage. However, a review of the data led to the emergence of factors associated with child marriage. There were no effective factors in the search process, so many studies that investigated effective factors were missed, yet this study can be a good guide for future researchers.

## Conclusion

The present study showed that the prevalence of child marriage is high in many regions of Africa and South Asia. Although interventions to reduce child marriage are underway and the trend of child marriage is decreasing, further acceleration is needed to achieve the 2030 development goals. Also, the present study identified the most important factors associated with child marriage, including low education, poor socio-economic status, rural residence, less developed nature of the place of residence, religions such as Islam, Hinduism and Christianity, a lack of access to media, and decision-making on marriage by parents. These factors are interconnected like a network, and it is essential to perceive the intricacies of these relationships.

Estimating the prevalence of child marriage and identifying the associated factors will enable researchers, policy makers, decision makers and health service providers to guide evidence-based planning to sooner and better achieve the Sustainable Development Goals. Empowering girls and parents, developing internet access, access to media, and reducing inequality and poverty are the most important solutions at regional and global levels.

### Supplementary Information

**Additional file 1:** **Table S1.** Inclusion and exclusion criteria. **Table S2.** Joanna Briggs Institute critical appraisal checklist for analytical cross sectional studies.

## Data Availability

All data related to this study are reported in this document.

## Abbreviations

UNICEF: United Nations Children’s Fund
CM: Child Marriage
UNICEF: United Nations Children’s Fund
UNFPA: United Nations Population Fund
STD: Sexually Transmitted Diseases
Mesh: Medical Subject Headings
