# Supplementary material for: Prevalence and factors associated with child marriage, a systematic review
Source: BMC Womens Health. 2023 Oct 10;23:531. doi: 10.1186/s12905-023-02634-3 (PMC10565969; doi:10.1186/s12905-023-02634-3)
Supplement: Supplementary file 1 — Additional file 1: Table S1. Inclusion and exclusion criteria. Table S2. Joanna Briggs Institute critical appraisal checklist for analytical cross sectional studies. [file 12905_2023_2634_MOESM1_ESM.docx]

**Table S1: Inclusion and exclusion criteria**

| Exclusion Criteria | Inclusion Criteria |  |
| --- | --- | --- |
| women and men get married higher 18 years of age  Special populations such as refugees, among whom child marriage is more prevalent | Population get (women) married under 18 years | Participants |
| Controlled trials, Quantitative designs, quasi experimental studies, and pre-test/post-test studies, reports ,letter to editor | Cross sectional studies  All studies that reported the prevalence of child marriage | Study Type |
|  | No intervention | Intervention |
| Refugee camp, females  living in Roma settlements | Any type of socio-health setting | Setting |
|  | Prevalence of child marriage  Trend of child marriage  Assess effective factors | Outcomes |
| Scientific articles before 2000. | Scientific full text articles published in indexed scientific journals | Dissemination Type |
| Articles written in languages other than  English | English | Language |
|  |  | Others |

**Table S2**. Joanna Briggs Institute critical appraisal checklist for analytical cross sectional studies

|  | Critical appraisal checklist | Yes | No | Unclear |
| --- | --- | --- | --- | --- |
| 1 | Were the criteria for inclusion in the sample clearly defined? | 4 | - | 30 |
| 2 | Were confounding factors identified? | 16 | - | 18 |
| 3 | Was the exposure measured in a valid and reliable way? | 1 | 33 | - |
| 4 | Were the study subject and the setting described in detail? | 11 | - | 23 |
| 5 | Were strategies to deal with confounding factor stated? | 26 | - | 8 |
| 6 | Was appropriate statistical analysis used? | 26 | - | 8 |
